# Cognitive Function Impairments Linked to Alcohol and Cannabis Use During Adolescence: A Study of Gender Differences

**DOI:** 10.3389/fnhum.2020.00095

**Published:** 2020-04-07

**Authors:** Simasadat Noorbakhsh, Mohammad H. Afzali, Elroy Boers, Patricia J. Conrod

**Affiliations:** Département de Psychiatrie, Université de Montréal, Centre de Recherche du CHU Sainte-Justine, Montréal, QC, Canada

**Keywords:** cognitive function, alcohol, cannabis, gender difference, adolescent

## Abstract

Major neurocognitive changes occur during adolescence, making this phase one of the most critical developmental periods of life. Furthermore, this phase in life is also the time in which youth substance use begins. Several studies have demonstrated the differential associations of alcohol and cannabis use concerning the neurocognitive functioning of both males and females. Past and contemporary literature on gender-specific effects in neuroscience of addiction is predominantly based on cross-sectional datasets and data that is limited in terms of measurement variability. Given the importance of gender-specific effects in addiction studies, and in order to address the two above-mentioned gaps in the literature, the present study aimed to compare neurocognitive functioning of male and female adolescents in the context of cannabis and alcohol use, while employing a longitudinal design with multiple repeated measurements. Participants were 3,826 high school students (47% female; mean age, 12.7), who were recruited from 31 high schools in the greater Montreal area. Participants were requested to complete annual surveys for five consecutive years, from 7th to 11th grade, assessing their alcohol/cannabis use and neurocognitive functioning (working memory, delayed recall memory, perceptual reasoning, and inhibition control). The analytical strategy focused on the longitudinal association between each predictor (female, male) and each of the outcomes (domains of neurocognitive functioning). Multilevel linear models assessed the association of alcohol and cannabis consumption and the four domains of neurocognitive functioning. Results revealed a gender by within-subject interaction, suggesting a weaker effect of yearly fluctuation of cannabis use on working memory among males compared to females. Our findings suggest a different pattern of neurocognitive impairment of female and male working memory after using cannabis over the course of adolescence. Early initiation of cannabis use potentially results in more spatial working memory deficits in female adolescents. This may negatively influence young females’ capacity in academic settings and lead to significant impairment in adulthood, which critically decreases the individual’s quality of life.

## Introduction

Given the increased rate of substance use from early to late adolescence (Duncan et al., 2006), it is becoming more and more critical to understand the effects of substance use on teens’ neurocognitive functioning. Alcohol and cannabis are the most commonly used psychoactive substances in Canada (Statistics Canada, 2015). Heavy drinking during adolescence has been indicated as a significant factor for declined memory (Mahmood et al., 2010) and impaired neurocognitive functioning (Mahmood et al., 2010), while cannabis use has been demonstrated to be associated with short-term and long-term cognitive deficits, such as impaired inhibitory control and working memory (Volkow et al., 2016; Morin et al., 2019). The proportion of males aged 12 years and over using alcohol or cannabis is approximately 5% to 10% higher than that of females in the same age group (Leatherdale and Burkhalter, 2012; Statistics Canada, 2018). Although the rate of substance use is different for male adolescents than for female adolescents, contemporary knowledge concerning gender-specific trajectories of substance use is limited. In particular, research distinguishing between different neurocognitive outcomes attributed to alcohol and cannabis use in adolescence, as well as taking into account potential gender-specific varying effects, is scarce.

The developmental phase of adolescence is, among others, marked by a multitude of neurocognitive and psychosocial changes, making the phase of adolescence one of the most critical developmental periods of life (Giedd, 2015). Furthermore, experimentation with substance use often starts in adolescence and so does the process of addiction (Volkow et al., 2016). For example, more than 90% of people who have an addiction today started to use various substances before they were 18 years old (Public Health Agency of Canada, 2018). This could reflect normal adolescent-specific behaviors (risk-taking, novelty-seeking, response to peer pressure) that increase the probability of someone experimenting with substances, and perhaps could also reflect the incomplete development neurocognitive functioning (Sowell et al., 2004).

The latter has been demonstrated by previous work showing that, relative to young adults and older people, the balance between adolescents’ reward motivation and executive control is not fully developed, therefore making adolescents more prone to engaging in health-risk behaviors such as alcohol use and cannabis use (Hammond et al., 2014). This disturbed balance has also been shown to accentuate the difference between adolescents who frequently engage in health-risk behaviors and those who do not (Squeglia et al., 2009). However, this differing balance has not only been found between adolescents and older people, and adolescents who frequently engage in health-risk behavior and those who do not, but also between male and female adolescents, predominantly because male and female adolescents do not share the same brain structure and neurodevelopmental pace (Lenroot and Giedd, 2010). That is, it has been suggested that early exposure to alcohol and cannabis use affects male and female adolescents’ neurocognitive development differently. Therefore, identifying gender-specific influences of alcohol and cannabis use separate for male and female adolescents could be beneficial to explain differential proneness to substance use in adolescents. Although, while within the realm of research on substance use, the importance of standard reporting on gender differences has been well acknowledged, only one-fourth of all studies on adolescent substance use have reported on this (Karlsson Lind et al., 2017). Thus, the results of this longitudinal study could potentially contribute in moving a step forward within this specific field of research.

To date, several studies have demonstrated differential associations of alcohol use, brain structure, and neurocognitive functioning for male and female adolescents. Specifically, Medina et al. (2008) examined the role of gender concerning the association of alcohol-use disorder and prefrontal cortex (PFC) morphometry in adolescents. Despite similar patterns of alcohol use, and even after controlling for variables such as conduct disorder and family history of substance-use disorders, Medina and colleagues found that gender moderated the association of alcohol-use disorder and PFC morphometry in adolescents. Also, it was revealed that, compared to same-gender controls, females showed smaller volumes of PFC morphometry, whereas males showed larger volumes (Medina et al., 2008). These findings are in line with previous work on functional neuroimaging, reporting that males suffering from alcohol-use disorder had increased superior frontal activation while female drinkers had limited superior frontal activation during spatial working memory tasks (Caldwell et al., 2005). The latter indicates, and has been supported by other works, that the fronto-parietal network regions could be particularly susceptible to alterations due to alcohol misuse/use, with females portraying greater adverse effects than males (Caldwell et al., 2005; Squeglia et al., 2012). Moreover, it has been proposed that regions in the brain network develop sooner among females than males (Giedd et al., 1996), implying that females may experience a stronger impaired working memory than males, if alcohol use has its onset in early adolescence (Wager and Smith, 2003).

Another brain region that might show a differing developmental trajectory for male and female adolescents when it concerns substance use is the PFC. The PFC has been shown to have protracted development and has been identified to be the last region of the brain to develop in adolescence. Several pre-clinical studies suggested that exposure to cannabis products during adolescence impacts neuromaturational processes in this region (Miller et al., 2019). Furthermore, functional neuroimaging studies found abnormal PFC activation patterns among adolescent marijuana users compared to controls, when it concerned an inhibition related go/no-go task (Tapert et al., 2007), as well as verbal memory (Jacobsen et al., 2007) and spatial working memory (Schweinsburg et al., 2008) tasks. Despite these valuable study results, when it concerns the moderating role of gender on the association of PFC structure and function and cannabis use in adolescents, past and contemporary findings are rather inconsistent. Whereas Pfefferbaum et al. (2002) found increased myelination of the PFC among young women, another study by Nagel et al. (2006) revealed contrasting results. Specifically, Nagel et al. (2006) found that women had reduced PFC white matter volume than men, of which the white matter volume of men remained moderately unaffected. Finally, in a study of PFC morphology, Medina et al. (2009) reported that, after 28 days of abstinence, female cannabis users showed higher volumes of PFC as well as a poorer performance on executive functioning tasks, whereas the control group demonstrated the opposite pattern.

To date, there is a strong body of research on the potential consequences of alcohol and cannabis use on brain structure and cognitive function in clinical, adult populations (Adger and Saha, 2013; Kuntsche and Gmel, 2013; Volkow et al., 2016). However, many previous studies utilized cross-sectional designs, which do not allow for causal modeling of associations (McHugh et al., 2018). However, to our knowledge, there is one notable exception. Using a longitudinal design, Morin et al. (2019) investigated the time-varying association of substance use (cannabis and alcohol) and neurocognitive functioning (inhibition control, perceptual reasoning, working memory, and delayed recall memory). The result of this study demonstrated that cannabis use has potential neurotoxic effects on inhibitory control and working memory of all the participants (Morin et al., 2019). Although, we value the study of Morin et al. (2019), they did not take into account the role of gender, which is rather striking given the previously presented work on the differences concerning neurocognitive functioning between female and male adolescents. Therefore, in extending the work by Morin et al. (2019), the present study aimed to explore potential differences in male and female adolescents concerning the development of neurocognitive functions in the context of alcohol and cannabis use over the course of adolescence.

In doing so, while also extending previous and contemporary cross-sectional works, we developed a longitudinal study in which we compared male and female adolescent neurocognitive functioning (i.e., working memory, recall memory, perceptual reasoning, and inhibitory control) in the context of alcohol and cannabis. We analyzed this prospective data using a multi-level statistical framework allowing for the dissociation of three different, yet potentially additive (or interacting), associations of low neurocognitive functioning and substance use: common vulnerability, time-varying concurrent (same year) relationships, and time-varying lagged relationships. Based on previous works on the different levels of vulnerability of females and males to substance use in samples of adults and adolescents (Medina et al., 2008; Squeglia et al., 2009, 2011, 2012; Alfonso-Loeches et al., 2013; Ewing et al., 2014; McHugh et al., 2018), we hypothesized that there is a difference between neurocognitive functioning of males and females linked to alcohol and cannabis use over the course of adolescence.

## Materials and Methods

### Participants

Participants were 3,826 high school students [47% female; mean age, 12.7 years (SD = 0.5)] from the Co-Venture study (NCT01655615; Landry et al., 2004; O’Leary-Barrett et al., 2017). A more detailed description of this study has been published elsewhere (O’Leary-Barrett et al., 2017). Participants were recruited from 31 public or private (French/English) high schools in the greater Montreal area, and were requested to participate in annual surveys for five consecutive years, from 7th to 11th grade. Among others, those surveyed had their alcohol and drug use, neurocognitive functions, and personality dimensions assessed. Our sample of high school students consisted of 15% of the entire population of 7th grade high school students in the greater Montreal area and they epidemiologically matched the size and socioeconomic status of each school district. Participant inclusion criteria consisted of providing informed assent and parent consent. Participants were excluded if they had unusual response patterns (e.g., same answer, sham drug item) or were reacting faster than usual (Reaction Time). Among the participants who completed the annual surveys, 3,659 (95.6%) of them were included in the analysis based on the minimal response to the questions and demographic information. The Co-Venture study obtained ethical approval from the ethics committee of the Sainte-Justine Hospital and the school boards of the schools that were recruited.

### Measures

Substance use and disorders (alcohol and cannabis) were evaluated by the modified version of the “Detection of Alcohol and Drug Problems in Adolescents” questionnaire (Landry et al., 2004). Participants were asked to rate the frequency of their substance consumptions on a scale of 0–5 (never to everyday). There was a specific question for the quantity of alcohol consumed, but not for cannabis consumption. In line with previous studies in the field of substance use, assessing the quantity of used cannabis is still a challenge (Piontek et al., 2008).

More details regarding the frequency and quantity of alcohol use and frequency of cannabis use can be found in Tables s 1, 2. Self-reports measuring substance use during adolescence can be more accurate than biological measures (such as urine tests) when the confidentiality is guaranteed (Clark and Winters, 2002), as there is a higher chance of reporting any episodic substance use. In the Co-Venture study, confidentiality was guaranteed unless there was a risk of harm to self or others.

**Table 1:** Frequency distribution for substance use variables in females over 5 years.

Substance and assessment for girls^a^		Frequency or quantity				
Frequency	Never	Occasionally	Once a month	Once or twice per week	Three times or more per week	Every day
Cannabis use
Year 1	47.19%	1.27%	0.21%	0.11%	0.08%	0.08%
Year 2	37.51%	2.67%	0.82%	0.50%	0.24%	0.16%
Year 3	30.44%	4.45%	1.14%	1.43%	0.42%	0.32%
Year 4	26.39%	6.56%	1.51%	1.11%	0.48%	0.56%
Year 5	21.65%	8.02%	2.09%	1.75%	0.48%	0.42%
Alcohol use
Year 1	33.35%	13.79%	1.06%	0.66%	0.03%	0.05%
Year 2	20.14%	17.73%	2.86%	1.03%	0.13%	0.00%
Year 3	12.78%	18.08%	4.79%	2.46%	0.05%	0.03%
Year 4	7.89%	17.76%	6.99%	3.79%	0.16%	0.03%
Year 5	5.24%	15.96%	7.62%	5.29%	0.29%	0.00%
**Number of standard drinks on a drinking occasion**						
**Quantity^b^**	**<1**	**1–2**	**3–5**	**6–8**	**>8**	
Alcohol use
Year 1	2.65%	5.29%	1.27%	0.11%	0.08%	
Year 2	2.49%	9.40%	3.10%	0.50%	0.21%	
Year 3	1.59%	11.86%	5.69%	1.03%	0.40%	
Year 4	1.40%	11.86%	9.11%	2.17%	0.56%	
Year 5	0.85%	11.41%	11.59%	2.33%	0.48%	

*^a^Year 1: assessment in 7th grade, year 2: 8th grade, and so on. ^b^Alcohol use quantity variables were categorized here for presentation purposes; in the analyses, alcohol use quantity was used as a continuous variable*.

**Table 2:** Frequency distribution for substance use variables in males over 5 years.

Substance and assessment for boys^a^		Frequency or quantity				
Frequency	Never	Occasionally	Once a month	Once or twice per week	Three times or more per week	Every day
Cannabis use
Year 1	47.41%	1.48%	0.53%	0.34%	0.24%	0.32%
Year 2	38.22%	2.57%	0.50%	0.45%	0.16%	0.16%
Year 3	31.18%	4.95%	0.64%	0.93%	0.45%	0.58%
Year 4	25.36%	6.51%	1.16%	1.43%	0.77%	1.01%
Year 5	19.90%	6.91%	2.22%	1.83%	1.06%	1.24%
Alcohol use
Year 1	29.27%	18.03%	1.99%	0.74%	0.16%	0.13%
Year 2	20.12%	17.68%	3.18%	0.87%	0.13%	0.08%
Year 3	14.06%	18.10%	4.21%	2.12%	0.16%	0.08%
Year 4	9.16%	15.11%	6.75%	4.61%	0.37%	0.24%
Year 5	6.14%	12.73%	6.70%	6.88%	0.53%	0.19%
**Number of standard drinks on a drinking occasion**						
**Quantity^b^**	**<1**	**1–2**	**3–5**	**6–8**	**>8**	
Alcohol use
Year 1	4.42%	6.62%	1.16%	0.29%	0.21%	
Year 2	4.02%	8.52%	1.88%	0.42%	0.26%	
Year 3	3.02%	10.01%	4.10%	0.98%	0.48%	
Year 4	1.80%	10.03%	7.41%	2.99%	0.56%	
Year 5	1.14%	8.58%	8.71%	4.16%	1.32%	

*^a^Year 1: assessment in 7th grade, year 2: 8th grade, and so on. ^b^Alcohol use quantity variables were categorized here for presentation purposes; in the analyses, alcohol use quantity was used as a continuous variable*.

### Outcomes

Utilizing a computerized neuropsychological assessment battery, the following cognitive functions were assessed. The detailed description of measures can be found in the original study protocol (O’Leary-Barrett et al., 2017).

Spatial working memory: like the spatial working memory sub-test of the Cambridge Neuropsychological Test Automated Battery (Owen et al., 1990), “Find the Phone” task was the measurement tool for assessing spatial working memory. This task is based on the Self-Order Pointing Task (Cragg and Nation, 2007) and the subjects are asked to search through a number of phones which are supposed to ring. The measure of spatial memory deficit is the number of times that the participant reselects the items that have already rung. The task had good internal reliability, with Cronbach α coefficient of 0.88 (Cragg and Nation, 2007).

Delayed recall memory: to assess the delayed recall memory, the computerized version of the “Dot Location” test as a part of Child Memory Scales (Cohen, 1997) was used. In this task, the participants memorize the location of circles in eight different colors on the screen. Thirty minutes later, the subjects are asked to relocate the circles as they were placed on the previous image. Test-retest reliability ranged from 0.71 to 0.91 for subscales (Cohen, 1997).

Perceptual reasoning: to measure perceptual reasoning, an abbreviation of the original Cattell’s Culture Fair Intelligence Test was used. In this nine-item task, the adolescents were asked to complete a series of puzzles with an increasing level of difficulty (Bilker et al., 2012). The scores from this test are highly correlated with that of Raven’s 60-item perceptual reasoning matrices, with the correlation of 0.98 for the short form (Bilker et al., 2012).

Inhibitory control: to assess the cognitive control and response inhibition, an adopted version of Go/No-Go PALP (Passive Avoidance Learning Paradigm), which requires individuals to inhibit a rewarded response in order to prevent further punishment (Newman et al., 1985; Castellanos-Ryan et al., 2011), was used. By trial and error, subjects learn to react to “good” numbers and not react to “bad” numbers. The poorer response inhibition is the number of errors on trials involving a No-Go response. Confirming the previous studies, response inhibition is correlated with other functional imaging measures of PFC activities in Go-No-Go tasks (Whelan et al., 2012).

We controlled for socioeconomic status measured by the family affluence scale (Currie et al., 1997) and school-cluster effects in all of our analysis.

### Statistical Analysis

The analytic strategy was focused on the longitudinal association between each predictor (female, male) and each of the outcomes (domains of cognition). Multilevel linear models assessed the association of alcohol (quantity by frequency) and cannabis (frequency) consumption and the four domains of cognition (working memory, delayed recall memory, perceptual reasoning, and inhibitory control). Two separate multilevel linear models were estimated for longitudinal effects of cannabis and alcohol as time-varying predictors of perpetration. The levels were time (nested in individuals) and individuals (nested in schools). The time parameter was coded from one to five (the survey waves). Predictors were person-mean centered. For both outcomes, the predictor terms were as follows: gender, socioeconomic status, linear and quadratic effects of time, between-subject differences in consumption measured by average substance use (alcohol or cannabis) over all waves, within-subject difference in consumption measured by current year change in use with regards to participant’s mean use, and lagged within-subject measured by past year change in use with regards to participant’s mean use. As the results of these effects were reported in a previous publication (Morin et al., 2019): interaction of gender by average use over all assessments, interaction of gender by change in use current year compared with the participant’s mean use, and interaction of gender by past year’s substance use compared with the participant’s mean use. Between-subject effects were interpreted as a common vulnerability between consumption and poor neurocognitive performance, while within-subject effects were interpreted as potentially neurotoxic effects of substance use. The interaction of gender with within-person effects were interpreted as a potential sensitivity in one gender relative to the other with respect to the neurotoxic effects of substances on cognitive development. The intraclass correlation coefficient (ICC) function from the psych package in the R statistical environment was used to estimate the within-subject stability of cognitive data over time; ICCs were 0.74 for working memory, 0.80 for perceptual reasoning, 0.58 for delayed memory recall, and 0.68 for response inhibition.

## Results

Overall, 3,826 students [2,028 boys (53%); mean age, 12.7 years] were involved. Analyses included the interactions of cannabis/alcohol use and gender, time, and SES. For socioeconomic status, the participants with lower SES revealed worse perceptual reasoning. Considering the main variables, the quantity of alcohol use and the frequency of cannabis use increased yearly for both genders (Tables s 1, 2).

### Cannabis Model

Table 3 presents results for the cannabis model. The results indicated a significant between-person effect of cannabis (the general level of cannabis use) on inhibition control (*β* = 2.10, SE = 0.71, *p* = 0.001). Furthermore, it was shown that the past year fluctuation in cannabis use was significantly associated with females’ perceptual reasoning (*β* = 0.12, SE = 0.05, *p* = 0.02). When we included the interaction with male-gender in our model, cannabis use revealed differential association of cannabis use and working memory among genders (β = −0.51, SE = 0.25, *p* = 0.04), implying potentially different neurotoxic effects of cannabis use for male and female adolescents. There were no significant interactions between time and gender, or time and gender and cannabis use (Supplementary Table S1).

**Table 3:** Estimated parameters for cannabis model in a school sample of adolescents assessed over 5 years^a^.

	Working memory			Perceptual reasoning			Delayed recall memory			Inhibition control		
	Estimate	SE	*p*	Estimate	SE	*p*	Estimate	SE	*p*	Estimate	SE	*p*
Intercept	21.04	1.50	0.00	15.48	0.42	0.00	17.86	0.48	0.00	34.80	3.00	0.00
Time	−6.76	0.97	0.00	1.25	0.27	0.00	−9.58	0.32	0.00	−8.92	1.96	0.00
Time squared	0.83	0.16	0.00	−0.10	0.04	0.02	2.00	0.05	0.00	0.88	0.33	0.01
SES*	0.10	0.08	0.20	−0.06	0.02	0.03	−0.03	0.02	0.16	0.31	0.15	0.04
Gender (female)	1.22	0.61	0.05	0.07	0.19	0.72	0.10	0.16	0.51	1.93	1.16	0.10
Cannabis, B*	0.23	0.37	0.54	−0.18	0.12	0.11	−0.08	0.10	0.42	**2.10**	**0.71**	**0.00**
Cannabis, W*	0.05	0.18	0.79	0.00	0.05	0.92	−0.09	0.06	0.12	−0.40	0.38	0.29
Cannabis, W (lagged)	−0.23	0.18	0.20	**0.12**	**0.05**	**0.02**	0.11	0.06	0.05	−0.13	0.38	0.73
Gender (male) × cannabis, B	0.25	0.49	0.61	−0.09	0.15	0.56	−0.01	0.13	0.93	−0.35	0.96	0.72
Gender (male) × cannabis, W	**−0.51**	**0.25**	**0.04**	−0.05	0.07	0.52	0.08	0.08	0.32	0.02	0.53	0.97
Gender (male) × cannabis, W (lagged)	0.40	0.25	0.12	0.00	0.07	0.98	−0.09	0.08	0.25	0.22	0.54	0.69

^a^Significant effects are indicated by boldface. Performance on working memory and inhibitory control tasks was measured by counting number of errors; a lower score indicates a better performance. *SES, socioeconomic status; B, between-subjects; W, within-subjects. Bold values correspond to significant predictors.

### Alcohol Model

Table 4 presents results for the alcohol model. When including the interaction with male-gender, the results indicated that alcohol use did not significantly interact with any of the neurocognitive domains. However, at the lagged-person level, it was shown that past year fluctuations in alcohol use were significantly associated with female adolescents’ inhibition control (*β* = −0.80, SE = 0.35, *p* = 0.02). Furthermore, at the between-person level, it was shown that alcohol use (general level of alcohol use) was not significantly associated with any of the neurocognitive domains when it concerned female adolescents.

**Table 4:** Estimated parameters for alcohol model in a school sample of adolescents assessed over 5 years^a^.

	Working memory			Perceptual reasoning			Delayed recall memory			Inhibition control		
	Estimate	SE	*p*	Estimate	SE	*p*	Estimate	SE	*p*	Estimate	SE	*p*
Intercept	21.54	1.49	0.00	15.12	0.42	0.00	17.71	0.48	0.00	37.80	2.97	0.00
Time	−6.50	0.99	0.00	1.18	0.27	0.00	−9.57	0.33	0.00	−9.41	1.98	0.00
Time squared	0.79	0.17	0.00	−0.09	0.05	0.04	2.00	0.05	0.00	0.95	0.33	0.00
SES*	0.12	0.08	0.15	−0.07	0.03	0.01	−0.04	0.02	0.05	0.38	0.16	0.02
Gender (female)	1.74	0.47	0.00	0.24	0.15	0.11	0.23	0.12	0.06	1.21	0.92	0.19
Alcohol, B*	−0.41	0.32	0.20	−0.03	0.10	0.74	0.06	0.08	0.49	0.22	0.61	0.72
Alcohol, W*	−0.20	0.18	0.26	0.08	0.05	0.13	0.03	0.06	0.62	0.28	0.35	0.42
Alcohol, W (lagged)	−0.10	0.18	0.57	0.02	0.05	0.75	−0.03	0.06	0.66	**−0.80**	**0.35**	**0.02**
Gender (male) × alcohol, B	0.62	0.43	0.15	0.00	0.14	0.97	0.00	0.11	0.97	0.28	0.85	0.74
Gender (male) × alcohol, W	0.12	0.24	0.61	−0.12	0.07	0.08	0.00	0.08	0.98	−0.16	0.49	0.75
Gender (male) × alcohol, W (lagged)	0.03	0.24	0.90	0.08	0.07	0.23	0.04	0.08	0.59	0.67	0.50	0.18

^a^Significant effects are indicated by boldface. Performance on working memory and inhibitory control tasks was measured by counting number of errors; a lower score indicates a better performance. *SES, socioeconomic status; B, between-subjects; W, within-subjects. Bold values correspond to significant predictors.

### Combined Alcohol-Cannabis Model

Table 5 presents the results of an integrated model of the simultaneous effect of alcohol and cannabis. The results revealed a male-gender by within-subject interaction, suggesting that the effect of yearly cannabis use fluctuation on working memory among males compared to females is weaker (*β* = −0.65, SE = 0.26, *p* = 0.01), meaning that females make more errors in working memory task than males. Furthermore, at the between-person level, it was revealed that alcohol use (general level of alcohol use) was significantly associated with perceptual reasoning (*β* = −0.94, SE = 0.38, *p* = 0.01) and inhibition control (*β* = −1.73, SE = 0.73, *p* = 0.02) of female adolescents only. Regarding the general level of cannabis use, the models revealed significant between-person associations of cannabis use and inhibition control, for female adolescents only (*β* = 3.06, SE = 0.86, *p* = 0.00). In addition, the past year fluctuation of cannabis use was shown to be significantly associated with female adolescents’ delayed recall memory (*β* = 0.12, SE = 0.05, *p* = 0.02).

**Table 5:** Estimated parameters for combined alcohol-cannabis model in a school sample of adolescents assessed over 5 years^a^.

	Working memory			Perceptual reasoning			Delayed recall memory			Inhibition control		
	Estimate	SE	*p*	Estimate	SE	*p*	Estimate	SE	*p*	Estimate	SE	*p*
Intercept	20.65	1.53	0.00	15.62	0.43	0.00	17.79	0.49	0.00	35.39	3.05	0.00
Time	−6.49	0.99	0.00	1.20	0.27	0.00	−9.56	0.33	0.00	−9.35	1.98	0.00
Time squared	0.80	0.17	0.00	−0.09	0.05	0.04	1.99	0.05	0.00	0.94	0.33	0.00
SES*	0.14	0.08	0.09	−0.08	0.03	0.00	−0.04	0.02	0.04	0.42	0.16	0.01
Gender (female)	0.96	0.67	0.15	0.10	0.21	0.62	0.17	0.17	0.32	1.68	1.29	0.19
Cannabis, B*	0.61	0.45	0.17	−0.27	0.14	0.06	−0.17	0.12	0.15	**3.06**	**0.86**	**0.00**
Cannabis, W*	0.14	0.18	0.45	−0.02	0.05	0.71	−0.11	0.06	0.08	−0.33	0.38	0.39
Cannabis, W (lagged)	−0.31	0.19	0.09	0.13	0.05	0.01	**0.13**	**0.06**	**0.03**	−0.22	0.38	0.57
Alcohol Frequency, B	**−0.94**	**0.38**	**0.01**	0.24	0.12	0.05	0.19	0.10	0.06	**−1.73**	**0.73**	**0.02**
Alcohol Frequency, W	−0.20	0.18	0.26	0.06	0.05	0.19	0.04	0.06	0.51	0.42	0.35	0.23
Alcohol Frequency, W (lagged)	−0.03	0.18	0.88	−0.02	0.05	0.63	−0.05	0.06	0.40	−0.67	0.36	0.06
Gender (male) × Cannabis, B	0.25	0.58	0.67	−0.10	0.18	0.57	0.03	0.15	0.86	−1.08	1.14	0.35
Gender (male) × Cannabis, W	**−0.65**	**0.26**	**0.01**	−0.03	0.07	0.68	0.09	0.08	0.29	−0.15	0.54	0.78
Gender (male) × Cannabis, (lagged)	0.50	0.26	0.06	−0.01	0.07	0.94	−0.10	0.08	0.22	0.33	0.55	0.55
Gender (male) × Alcohol, B	0.49	0.51	0.34	−0.02	0.16	0.89	−0.04	0.13	0.75	1.22	0.99	0.22
Gender (male) × Alcohol, W	0.32	0.24	0.19	−0.12	0.07	0.09	−0.02	0.08	0.84	−0.21	0.50	0.67
Gender (male) × Alcohol (lagged)	−0.09	0.25	0.73	0.09	0.07	0.20	0.06	0.08	0.44	0.64	0.51	0.21

^a^Significant effects are indicated by boldface. Performance on working memory and inhibitory control tasks was measured by counting number of errors; a lower score indicates a better performance. *SES, socioeconomic status; B, between-subjects; W, within-subjects. Bold values correspond to significant predictors.

**Figure 1:**
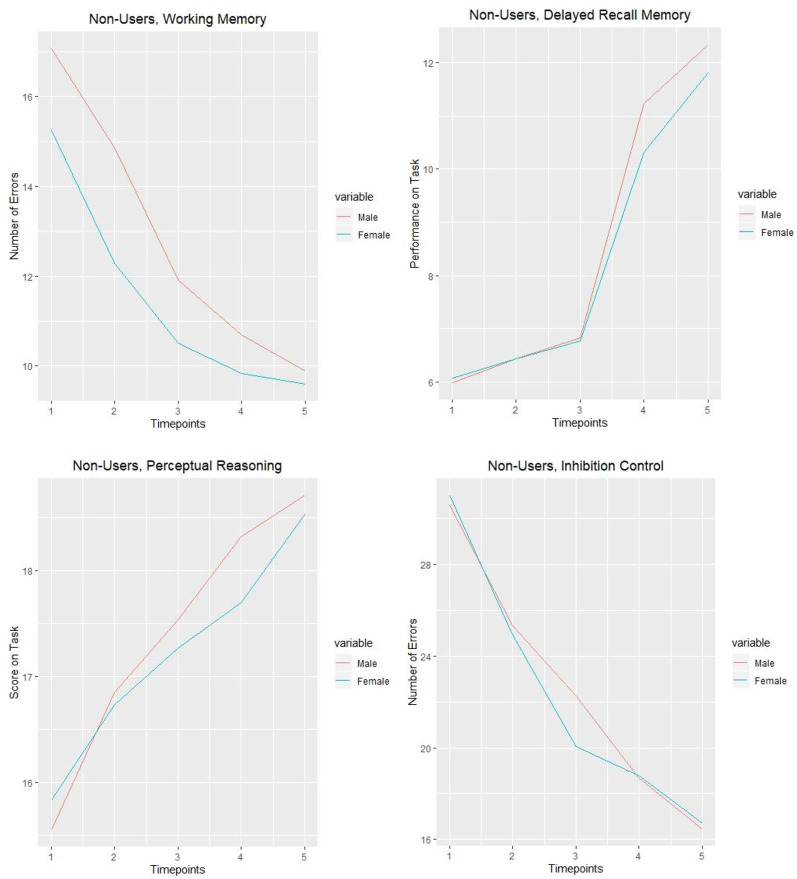
Non-user students’ cognitive tasks performance over 5 years (grade 7–11).

**Figure 2:**
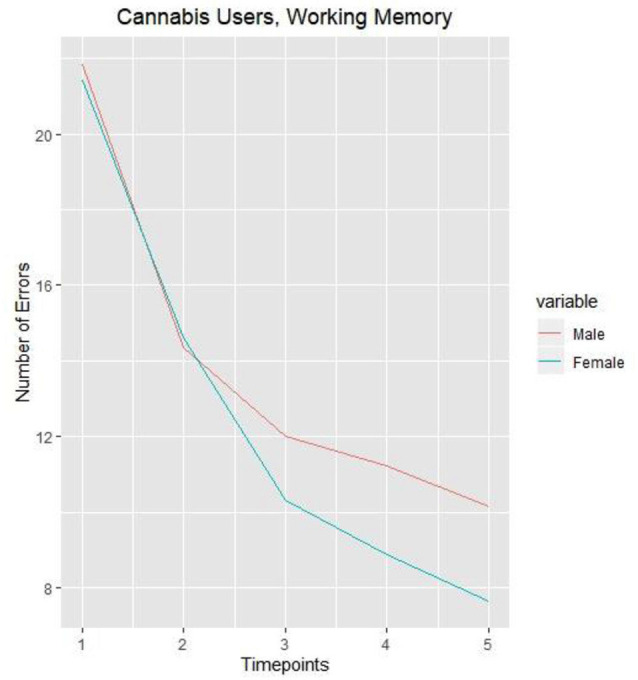
Number of errors on working memory task in male and female cannabis users (once a month and more) measured over 5 years.

To facilitate interpretation of these results, the mean of cognitive tasks performance of non-user adolescents are presented in Figure 1. In general, across all time points, non-using female adolescents were making fewer errors than boys during the working memory task. However, when it concerned the other cognitive tasks, no significant differences between male and female adolescents were observed. Furthermore, Figure 2 represents the working memory performance of those who were cannabis users and who used in a particular year. It was shown that female adolescents using cannabis displayed higher initial levels of errors concerning the working memory task than male adolescents using cannabis across time points 1 and 2, indicating that although non-cannabis using male adolescents made more errors during the working memory task, female cannabis users were shown to be more sensitive to the negative consequences of cannabis on working memory. However, these effects were shown to disappear over time.

## Discussion

This study examined the gender differences in female and male adolescents’ neurocognitive functioning (working memory, perceptual reasoning, delayed recall memory, and inhibition control) utilizing a longitudinal design among a large sample of nearly 4,000 North-American adolescents, distinguishing between three time-varying effects of predictor variables: between-person effect, within-person effect, and lagged within-person effect. Based on the results, several important conclusions can be drawn. First, among the studied neurocognitive functions, female cannabis users had significantly different levels of working memory impairments than males. We also found robust male/female differences in the combined model of alcohol and cannabis use, confirming the different effects of cannabis use on females and males working memory even after controlling for the effect of alcohol use. We did not observe such an effect for alcohol users. While significant alteration in the brain regions responsible for working memory have been reported in previous findings (Kanayama et al., 2004; Jager et al., 2006; Becker et al., 2010), results from this study revealed a new gender-specific developmental effect. Therefore, the gender-specific impairments related to cannabis use in females were limited to early stages of adolescent development, in line with the hypothesis that there are gender differences concerning the effects of cannabis on neurocognitive functioning in early adolescence.

Given the shifting policy of cannabis use laws, the prevalence of adolescents’ misuse/use is rising. Meanwhile, having a better understanding of the neurocognitive functions after exposure to cannabis for boys and girls separately is the key to leading future research on the optimal treatment methods for cannabis dependency. The current evidence on gender-specific underlying neurobiological mechanisms of executive functioning and decision making regions of the brain, can be a possible explanation for the “Telescoping” phenomenon, which narrates a faster progression from the first exposure to a substance to the addiction phase in women (Hernandez-Avila et al., 2004). To be more specific, working memory is an essential component in academic success at school (Aronen et al., 2005). At least 10% of females 15 years and above report using cannabis in the past year (Health Canada, 2018) which increases the risk of school drop out up to 2.3 times more than non-user students (Bray et al., 2000). Students’ cognitive function level decreases significantly for days after cannabis use (Crean et al., 2011) and for a considerable period, it affects their performance at school. In addition, the long term effects of cannabis on attention and memory are more long-lasting and severe when the individuals start using cannabis during adolescence (Schweinsburg et al., 2008) or are heavy-regular users (Solowij et al., 2002). Consequently, a secondary effect of acute intoxication, cannabis user students fail to learn at school, which in the long term leads to poorer grades and higher school drop out rates (Lynskey and Hall, 2000).

Working memory involves the ability to process and store information over a short time period and has been found to be predominantly associated with PFC and parietal cortex activities (van Asselen et al., 2006). In many studies, cannabis use was related to significant alterations in brain activity during functional magnetic resonance imaging (fMRI) tasks measuring spatial working memory (Jager et al., 2006; Becker et al., 2010). On the other hand, strong evidence on neurodevelopmental trajectories of the PFC shows discrepancy by gender. Due to sexual dimorphism during brain development, the full maturation process of female brain volumes is almost reached at the age of 10–11, while maturation could be as late as 14–15 years for male adolescents (Lenroot et al., 2007). Female PFC maturation peaks size 1 to 2 years earlier than for males (Giedd et al., 1996; Lenroot et al., 2007). As a result, females may experience more impairments in working memory than males, under the condition that cannabis use has its onset in adolescence.

Several studies have highlighted the importance of assessing the interaction between gender and age of onset after exposure to THC. In animal studies, while both male and female adolescent rats had impaired spatial working memory after cannabis exposure (O’Shea et al., 2006; Rubino et al., 2009), it was only the male rats with lasting memory deficit in adulthood (O’Shea et al., 2006). Also, in human subjects, gender can be a moderator in the association of brain structure, cognitive functioning, and cannabis use. For example, a number of studies highlighted that higher executive functioning (Medina et al., 2009) and memory performance impairments were linked to cannabis use (Gruber et al., 1997; Crane et al., 2013) in female adolescents. In contrast, as an acute effect of THC, Makela et al. found improved spatial working memory in young adult females (Makela et al., 2006). Those inconsistencies in the previous studies (Ketcherside et al., 2016) can be the result of differences in developmental stage, design of study (longitudinal/cross-sectional), levels of THC exposure and intoxication (Morin et al., 2019), and age of initiation (Gorey et al., 2019).

When considering gender differences in alcohol and cannabis effects on neurocognition, it is first important to account for the developmental sensitivity in neurocognitive performance. Considering the late maturation of brain substrates related to working memory among boys, there is a neuroplastic effect that decreases cannabis-related impairment among male adolescents compared to female adolescents. In contrast, as the maturation of prefrontal regions related to working memory happens earlier in girls, the negative effects of cannabis on working memory appears to be more pronounced during early adolescence. We can conclude that initiation of cannabis use during early adolescence might effect males and females differently due to these gender-based differences in neuromaturation (Lenroot and Giedd, 2010). Whether these drug-related changes are implicated in females’ elevated risk for substance use disorders is a question worthy of further investigation.

The current study has some limitations. First, we looked into the effects of alcohol and cannabis use, but not the substance use disorder as it is defined in the DSM-5 (American Psychiatric Association, 2013), or polysubstance use. As we did not have the clinical substance use data, the results from this study could not be generalized to clinical population. Second, like other studies on cannabis use, we could not identify the cannabis exposure quantity (Piontek et al., 2008). Cannabis legalization in North America might provide the opportunity to use a standard scale for cannabis intake in the future studies. Third, we applied a self-report scale for measuring alcohol and cannabis use and our assessment did not include more objective observation methods such as biological tests. Regarding the sensitive nature of reporting substance use, those behaviors might have been underreported. Fourth, even though cognitive functioning was assessed with valid and reliable instruments, the results could be different in clinical settings due to its limitations (e.g., false-positive/negative results, over-diagnosis; Roebuck-Spencer et al., 2017). As cognitive tests used in the current study were done in school and they were administrated with other tests, fatigue and boredom could affect the students’ cognitive functioning and neuropsychological status. In addition, we have not considered possible neurological or neurocognitive disorders of the participants. Finally, although observing the interaction of some other demographic variables such as SES (Johnson and Novak, 2009), sexual orientation (Medley et al., 2016), and racial/ethnic (Guerrero et al., 2014) differences with gender could be significant, this study was not intended to thoroughly explore those effects. Nevertheless, the current study was designed to report the association of gender differences and cognitive impairment due to alcohol and cannabis use during early ages.

In conclusion, the current study carried out one of the first analysis of gender differences in patterns of adolescents’ neurocognitive impairments, using a longitudinal design from the Co-Venture study across five consecutive years. The results from this study provide a more detailed understanding of gender-specific processes in addiction vulnerability that could be used to inform public health messaging and targeted drug and alcohol prevention for young people (Conrod, 2016). Spatial working memory deficits could negatively influence young females’ capacity in academic settings and could lead to significant impairment in adulthood, which critically decreases the individual’s quality of life.

## Data Availability Statement

The datasets generated for this study are available on request to the corresponding author.

## Ethics Statement

The studies involving human participants were reviewed and approved by Ethics Committee of Sainte-Justine Hospital and all administrative school board of involved high schools in Montreal. Written informed consent to participate in this study was provided by the participants’ legal guardian/next of kin.

## Author Contributions

SN and PC conceived the presented idea. MA developed the theory and performed the computations. PC verified the analytical methods. PC encouraged SN to investigate the cognitive developmental trajectories of male and female adolescents and supervised the findings of this work. EB critically reviewed and revised the manuscript. All authors discussed the results and contributed to the final manuscript.

## Conflict of Interest

The authors declare that the research was conducted in the absence of any commercial or financial relationships that could be construed as a potential conflict of interest.

## Supplementary Material

TABLE S1 Estimated parameters for cannabis model in a school sample of adolescents assessed over 5 years, interaction with TIME.
