# Corrigendum: Cognitive Function Impairments Linked to Alcohol and Cannabis Use During Adolescence: A Study of Gender Differences

**DOI:** 10.3389/fnhum.2020.00239

**Published:** 2020-07-15

**Authors:** Simasadat Noorbakhsh, Mohammad H. Afzali, Elroy Boers, Patricia J. Conrod

**Affiliations:** Département de Psychiatrie, Université de Montréal, Centre de Recherche du CHU Sainte-Justine, Montréal, QC, Canada

**Keywords:** cognitive function, alcohol, cannabis, gender difference, adolescent

In the original article, part of Figure 1 was included in Figure 2 by mistake. The corrected Figure 2 appears below.

**Figure 2:**
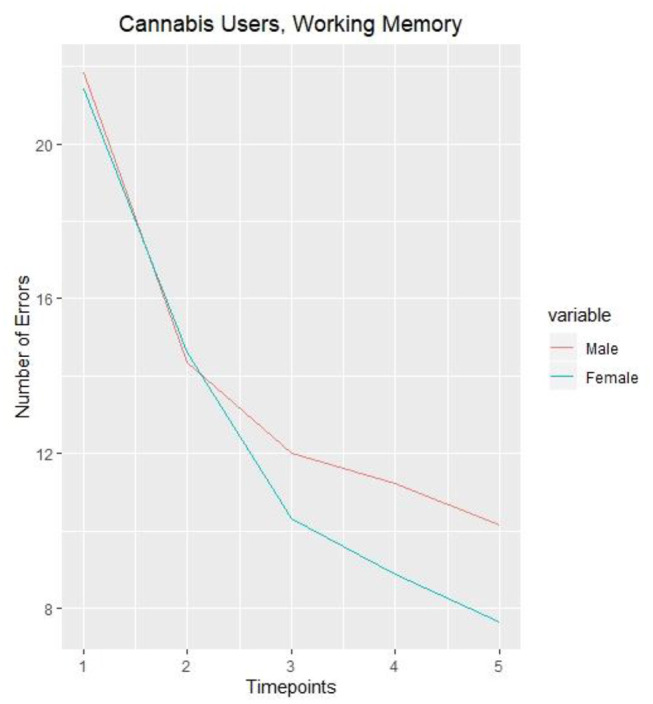
Number of errors on working memory task in male and female cannabis users (once a month and more) measured over 5 years.

The authors apologize for this error and state that this does not change the scientific conclusions of the article in any way. The original article has been updated.

